# Synthesis, characterization, and application of low-cost Mg-Al/CO_3_ and Ni-Al/CO_3_ layered double hydroxides (LDHs) as adsorbents for the removal of aniline blue dye from aqueous solutions: adsorption isotherms, kinetics, and thermodynamic studies

**DOI:** 10.1039/d5ra04856g

**Published:** 2025-08-04

**Authors:** Arman Ameen kaka Mohammed, Kareem Jumaah Jibrael Al-Salihi, Rebaz Fayaq HamaRawf

**Affiliations:** a Department of Chemistry, College of Science, University of Sulaimani Sulaymaniyah 46001 kurdistan region Iraq arman.kaka@univsul.edu.iq rebaz.hamarawf@univsul.edu.iq kareem.jibrael@univsul.edu.iq

## Abstract

Low cost Mg-Al/CO_3_ and Ni-Al/CO_3_ layered double hydroxides (LDHs) were prepared using a co-precipitation technique to improve their dye adsorption efficiency. Characterization techniques were employed to assess their structure and surface properties, including FT-IR, XRD, SEM, BET surface analysis, and UV-vis spectroscopy. The BET analysis revealed surface areas of 82.63 m^2^ g^−1^ for Mg-Al/CO_3_ and 5.95 m^2^ g^−1^ for Ni-Al/CO_3_, highlighting their porous nature. The materials' capability to adsorb aniline blue from water was tested under various conditions, such as time, pH, dye concentration, and temperature. The adsorption data were analyzed using both Langmuir and Freundlich isotherm models. The Mg-Al/CO_3_ LDH exhibited a notably higher maximum adsorption capacity of 704 mg g^−1^, compared to 181 mg g^−1^ for Ni-Al/CO_3_ LDH, according to the Langmuir model. The correlation coefficients (*R*^2^) indicate that the Freundlich model better fits the Mg-Al/CO_3_ LDH data (*R*^2^ = 0.9829), while the Langmuir model offers a better fit for Ni-Al/CO_3_ LDH (*R*^2^ = 0.9576). While kinetic studies showed that the pseudo-second-order model provided the best fit. The process was spontaneous and endothermic based on thermodynamic analysis, with positive entropy changes suggesting increased randomness at the interface. These results underscore the promise of using Mg-Al/CO_3_ and Ni-Al/CO_3_ LDHs as economical adsorbents in water purification.

## Abstract 2

Low cost Mg-Al/CO_3_ and Ni-Al/CO_3_ layered double hydroxides (LDHs) were prepared using a co-precipitation technique to improve their dye adsorption efficiency.

## Graphical abstract

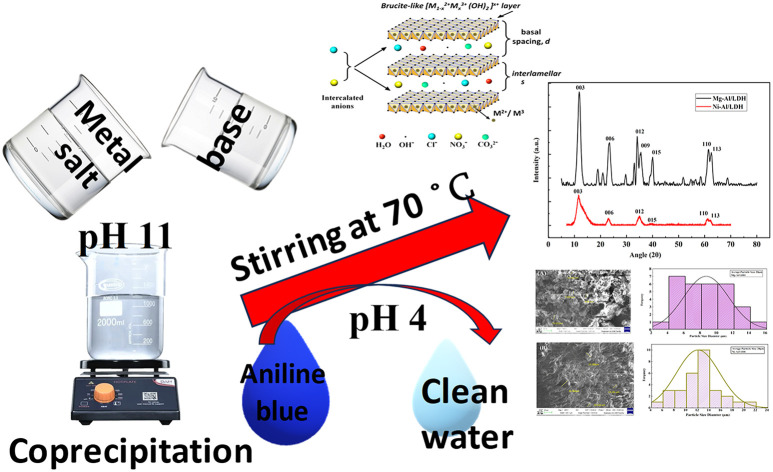

## 1 Introduction

Layered double hydroxides (LDHs) represent a class of anionic clay like materials characterized by their distinctive layered structures.^1^ These structures are composed of positively charged mixed metal hydroxide layers, interspersed with charge-balancing anions and water molecules.^2^ The general chemical formula of these layered double hydroxides can be written as:[M(ii)_1−*x*_M(iii)_*x*_(OH)_2_] (A^*n*−^)_*x*/*n*_·*m*H_2_Owhere, M(ii) is a divalent cation, M(iii) is a trivalent cation, A is an interlayer anion, *n*^−^ is a charge on the interlayer ion, and *x* and *y* are fraction constants.^3^ A significant feature of LDHs is their capacity to exchange interlayer anions while maintaining their layered framework.^4^ Type of materials are well known for their applications such as catalyst or catalyst supports^5^ ion exchangers^6^ biosensors based on clay modified electrodes,^7^ photoanode electrode in dye sensitized solar cells^8^ and also used as an adsorbent to remove dyes from effluents of textile, plastic and paper industries. Additionally, thermal treatment of LDHs can disrupt their layered structure, resulting in the formation of mixed metal oxides with high specific surface areas.^9^

Extensive research has focused on graphene-based nanomaterials due to their outstanding surface area, robust chemical stability, and excellent adsorption properties.^10^ These characteristics make them highly effective for eliminating contaminants from wastewater through mechanisms such as adsorption, photocatalysis, and electrochemical processes.^11^ Combining flocculation with photocatalysis presents a synergistic strategy for enhanced wastewater treatment efficiency.^12^ This hybrid approach has been effectively utilized in the removal of synthetic dye pollutants.^13^ The removal of synthetic dyes from wastewater is a critical environmental challenge due to their toxicity, non-biodegradability, and potential to harm aquatic ecosystems. Aniline blue, a triarylmethane dye, is widely used in textiles, paper, and biological staining but poses significant environmental risks.^14^ Layered double hydroxides (LDHs) have emerged as promising adsorbents for dye removal due to their unique structure, high surface area, and tunable properties.^15^ Various synthetic method uses for prepare LDH with different structure, composition and properties. The method commonly used are coprecipitation,^16^ hydrothermal,^17^ urea hydrolysis,^18^ ion-exchange,^19^ and sol- gel method.^20^ Each of this method offering clear advantage in term crystallinity purity and scalability for example coprecipitation method is widely use because its simplicity and ability to prepare LDH which have defined structure.^21^ In recent years, industrial grow have significance impact on environmental pollution especially on water pollutant such as organic dye^22^ for this problem have many deferent and efficient way to remove this pollution one of the most commonly method is adsorption.^23^ LDH one of the best adsorbents for removing organic dye in waste water.^24^ Recent studies have explored the modification of LDHs to enhance their catalytic properties for aniline degradation. For instance, Zhang *et al.*^24,25^ reported the synthesized Co/Al–LDH-coated silicon carbide membrane filters (SCMFs) to activate persulfate for aniline degradation. Their findings revealed that heating treatments improved the catalytic efficiency of these SCMFs, achieving approximately 95% aniline removal within 20 minutes under specific conditions.^25^ The objective of this project is to synthesize and characterize low-cost Mg-Al/CO_3_ and Ni-Al/CO_3_ layered double hydroxides (LDHs) and evaluate their effectiveness as adsorbents for the removal of aniline blue dye from aqueous solutions. The study aims to investigate the adsorption behavior through isotherm modeling, kinetic analysis, and thermodynamic evaluation to understand the mechanisms involved and assess the practical potential of these LDHs for wastewater treatment applications.

## 2 Experimental

### 2.1 Materials

All chemical reagents were used without further purification, Mg(NO_3_)_2_·6H_2_O, of Al(NO_3_)_3_·9H_2_O Ni(NO_3_)_2_·6H_2_O, NaHCO_3_ and NaOH, were purchased from Sigma Aldrich. Aniline blue dye (molecular formula: C_32_H_25_N_3_Na_2_O_9_S_3_ and formula weight is 737.74) purchased from Sigma.

### 2.2 Preparation of Mg-Al/CO_3_ layered double hydroxide

Mg-Al/CO_3_(LDHs) were synthesized *via* a co-precipitation method adapted from a reported procedure.^8^ Initially, a solution was prepared by dissolving (16.8 g) sodium bicarbonate (NaHCO_3_, 2 M) and (0.8 g) sodium hydroxide (NaOH, 0.2 M) in 200 mL of distilled water, adjusting the pH to 11. Separately, another solution was prepared by dissolving 46.15 g of magnesium nitrate hexahydrate (Mg(NO_3_)_2_·6H_2_O) and 22.5 g of aluminum nitrate nonahydrate (Al(NO_3_)_3_·9H_2_O) in 400 mL of distilled water, maintaining a Mg/Al molar ratio of 2 : 1 (all reagents were obtained from Aldrich.)

Both solutions were heated to 70 °C and then simultaneously combined through a Pyrex glass T-connector, carefully controlling the addition rate to maintain the pH at 11 under continuous stirring. After the addition was complete, the resulting slurry was stirred at 70 °C for an additional hour, then allowed to cool to room temperature and left to age for 24 hours. The precipitate formed was collected by filtration, thoroughly washed with distilled water, and dried at 70 °C. The obtained LDH powder was characterized using X-ray diffraction (XRD), scanning electron microscopy (SEM), Surface area (BET), and Fourier-transform infrared spectroscopy (FT-IR).

### 2.3 Preparation of Ni-Al/CO_3_ layered double hydroxide

The Ni-Al/CO_3_ layered double hydroxide (LDH) was synthesized *via* a coprecipitation method at constant pH. Initially, an alkaline solution was prepared by dissolving 16.8 g of sodium bicarbonate (NaHCO_3_, 2 M) and 0.8 g of sodium hydroxide (NaOH, 0.2 M) in 200 mL of distilled water, and the pH was adjusted to 11. In a separate step, two metal salt solutions were prepare 7.26 g of nickel nitrate hexahydrate (0.25 M) was dissolved in 100 mL of distilled water. And (3.75 g of aluminum nitrate (0.1 M) was dissolved in another 100 mL of distilled water). Both the alkaline and metal salt solutions were preheated to 70 °C. Using a Pyrex glass T-connector, the two solutions were simultaneously and gradually added together under continuous stirring, ensuring that the pH was consistently maintained at 11 throughout the mixing process. After the addition was complete, the resulting suspension was stirred continuously at 70 °C for one hour. The mixture was then allowed to cool to room temperature and left to age undisturbed for 24 hours. The resulting precipitate was separated by filtration, thoroughly washed with distilled water to remove impurities, and then dried at 70 °C. The final Ni-Al/CO_3_ LDH powder was characterized by various techniques, including X-ray diffraction (XRD), scanning electron microscopy (SEM), Brunauer–Emmett–Teller (BET) surface area analysis, and Fourier-transform infrared spectroscopy (FT-IR).

### 2.4 Calibration curve of aniline blue dye

**Fig. 1:**
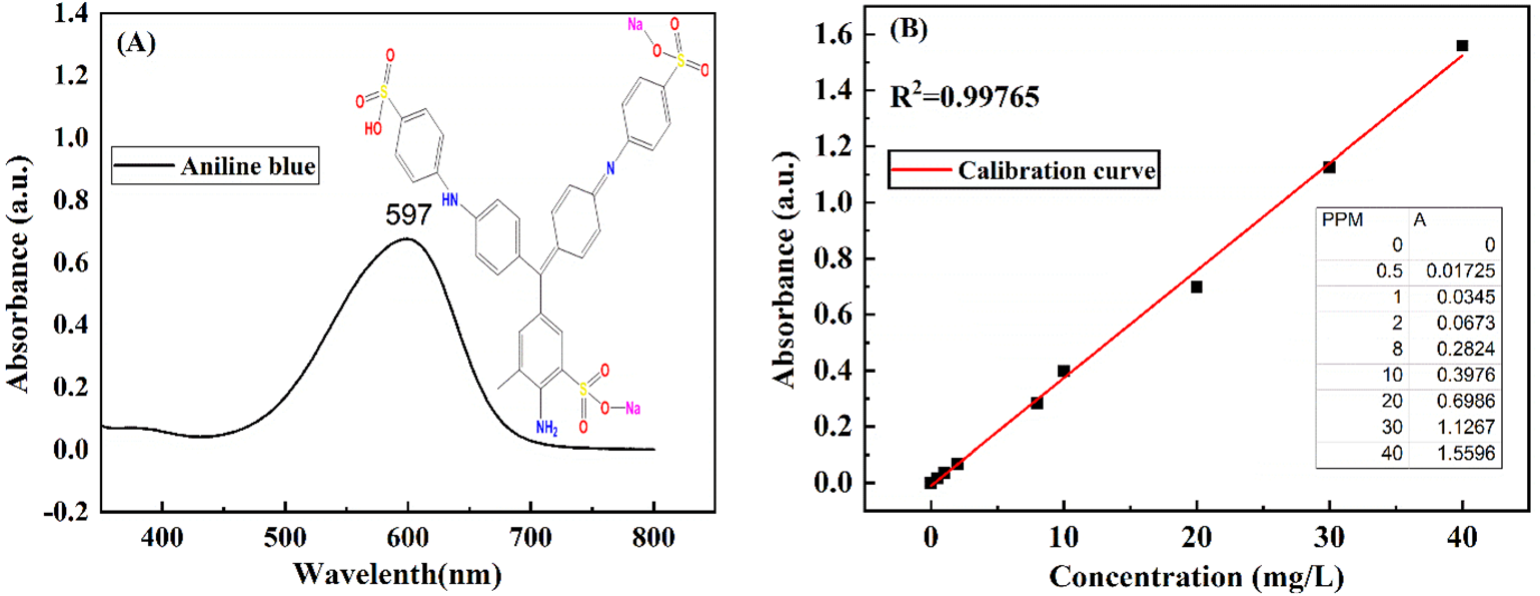
(A) The chemical structure and UV-vis spectrum of aniline blue dye (B) calibration curve of standard aniline blue dye solutions.

The prepared concentration of aniline blue dye in this study range between 0.5 -40 mg L^−1^ (ppm). Absorbance measurements were done by UV/vis spectrophotometer, the maximum absorbance (*λ*_max_) of aniline blue dye was determined by scanning a standard solution of known concentration at different wavelength as shown in Fig. 1A. Values of maximum absorbance was recorded and the (*λ*_max_) of aniline blue dye at 597 nm. The fixed wavelength was used for preparation of calibration curves shown in Fig. 1B.

### 2.5 Effects of contact time

The study investigated the impact of contact time on the sorption of aniline blue dye solution onto the surfaces of layered double hydroxide (Mg-Al/CO_3_ LDH) and Ni-Al/CO_3_ LDH at a constant temperature of 25 °C, and the absorbance of the dye was measured using UV/vis-spectroscopy at a fixed wavelength of 597 nm. This experiment was then repeated for the sorption of aniline blue dye onto adsorbents of LDHs at the same intervals. The amount of adsorbed dye was calculated according to the following equation.^26^1
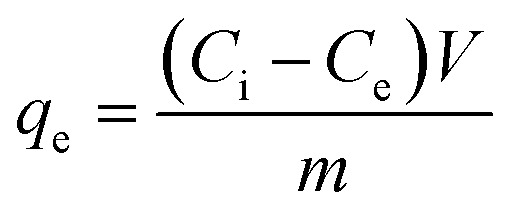
where, *q*_e_ amount of dye adsorbed (mg g^−1^), *C*_i_ initial concentration of adsorbed (mg L^−1^), *C*_e_ the concentration at equilibrium time (mg L^−1^), *m* mass of adsorbent (g) and *V* volume of dye solution (ml).

### 2.6 Characterization methods

#### 2.6.1 XRD

The XRD pattern was recorded using a Rigaku D/MAX-rA Xray diffractometer with Cu Kα radiation (*δ* = 0.15418 nm). The sample was scanned for 2*θ* values ranging from 3° to 70° with a scan speed of 1/min.

#### 2.6.2 FT-IR

FTIR spectrum was obtained using a Bruker Tensor 27 FT-IR Spectrometer where samples will be were finely ground and mixed with KBr and pressed into a disc. Spectrums of samples were scanned at 2 cm^−1^ resolution between 400 and 4000 cm^−1^.

#### 2.6.3 Scanning electron microscopy (SEM)

Scanning Electron Microscopy (SEM) was employed as a powerful method to analyze the surface morphology of Mg-Al/CO_3_ LDH and Ni-Al/CO_3_ LDH. To evaluate the effectiveness of the sorption process, SEM images of both LDH materials are shown in Fig. 4. As illustrated in the figure, hierarchical micro- and nanostructures developed, displaying various textural pores that became more prominent at higher magnifications. The morphology and particle size were influenced by the presence of cationic ions, which help minimize the surface energy disparity between polar and nonpolar crystal planes.

#### 2.6.4 BET measurement

The shape of the adsorption isotherm provides insight into the material's surface area. Both Mg-Al/CO_3_ LDH and Ni-Al/CO_3_ LDH exhibit predominantly mesoporous characteristics. Their BET surface areas were found to be 82.637 m^2^ g^−1^ for Mg-Al/CO_3_ LDH and 5.95 m^2^ g^−1^ for Ni-Al/CO_3_ LDH. Correspondingly, the pore volumes were measured at 0.435 cm^3^ g^−1^ and 0.033 cm^3^ g^−1^ for Mg-Al/CO_3_ LDH and Ni-Al/CO_3_ LDH, respectively.

UV-vis spectrometer ATI Unicom is typically utilized for the calculation of adsorbed aniline blue dye amounts on (Mg-Al/CO_3_ LDH) and Ni-Al/CO_3_ LDH. The absorbance spectrum of the remaining dye solution (after adsorption) is measured using UV-vis spectroscopy. By comparing this spectrum to the calibration curve established earlier, the concentration of dye molecules remaining in solution can be determined.

## 3 Results and discussion

### 3.1 Characterization of LDHs

**Fig. 2:**
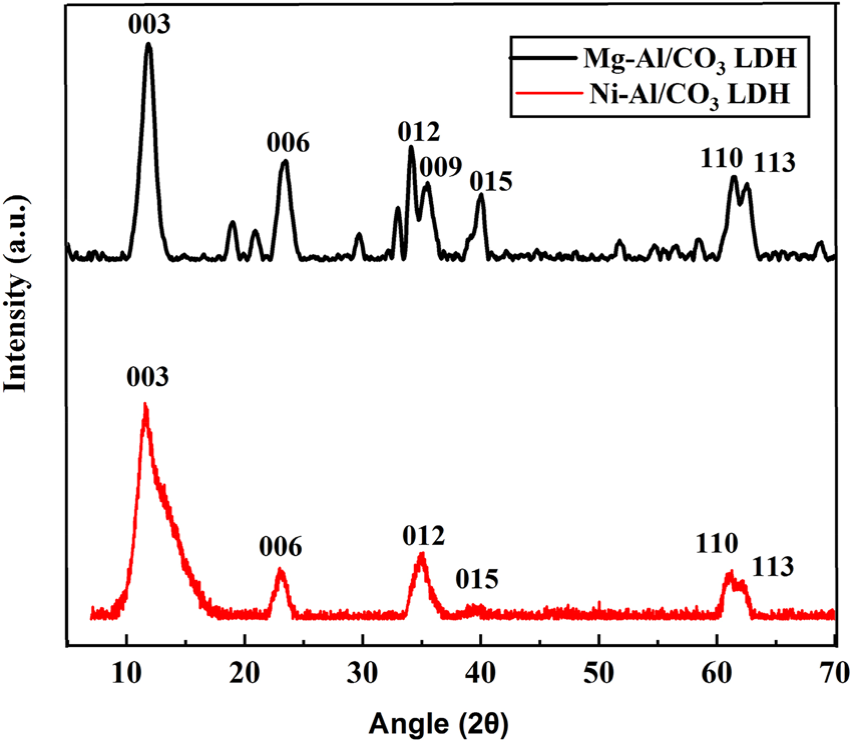
X-ray diffraction pattern of Mg-Al/CO_3_ LDH (black line) and Ni-Al/CO_3_ LDH (red line).

The Mg-Al/CO_3_ layered double hydroxide (LDH) was prepared using the co-precipitation technique, with its crystalline structure confirmed by the X-ray diffraction (XRD) pattern shown in Fig. 2, The diffraction data revealed a well-defined hydrotalcite-like LDH structure, characterized by prominent peaks at 11.52°, 23.53°, 35.13°, 39.81°, 47.14°, 61.28°, and 63.21°.^27^ Notably, strong and sharp reflections for the (003) and (006) planes at low 2*θ* angles of 11.52° and 23.53°, respectively, signified high crystallinity. Broader, asymmetric peaks at higher angles—especially around 35.13°—were also observed.^28^ The peak intensities, particularly those of the (003) and (006) planes, suggested differences in the crystallinity of the sample.^29^

Meanwhile he broader and weaker peaks at approximately 39.81° and 47.14° were assigned to the (012) and (015) planes. This explained as possibly impurity phases or overlapping reflections, not the same crystallographic planes. A distinct doublet near 61.28° and 63.23° corresponded to reflections from the (110) and (113) planes, respectively.^30^ The interlayer spacing (*d*-spacing) of the Mg-Al/CO_3_ LDH was calculated to be 7.56 Å, aligning with previous findings by Lee.^31^ The XRD analysis provides insights into the internal atomic arrangement of the material, as each crystal structure yields a distinct diffraction pattern based on the atomic configuration and spacing. By matching these patterns with known reference data, researchers can identify the phases present and infer structural characteristics of the synthesized compound.^28^

Fig. 2 shows the XRD pattern confirmed that the Ni-Al/CO_3_ layered double hydroxide (LDH) exhibits a well-ordered hydrotalcite-like structure, as evidenced by distinct peaks at 11.52°, 23.63°, 35.43°, 40.11°, 61.28°, and 63.21°, corresponding to the (003), (006), (012), (009), (015), (110), and (113) planes. The low-angle reflections, particularly (003) and (006), provide insight into the interlayer distances, with the sharp (003) peak at 11.52° indicating a highly structured layered arrangement and the presence of intercalated carbonate ions. The peaks (012), (009), and (015) reflect the organization of metal ions within the brucite-like layers and their vertical stacking pattern. In contrast, the high-angle peaks (110) and (113) are associated with the in-layer cation distribution and overall crystallinity. The clarity and intensity of these reflections indicate good structural order within the layers. These findings verify that the material forms a typical LDH lamellar architecture, featuring positively charged Ni-Al hydroxide sheets and carbonate anions situated in the interlayer gaps for charge neutrality.

**Fig. 3:**
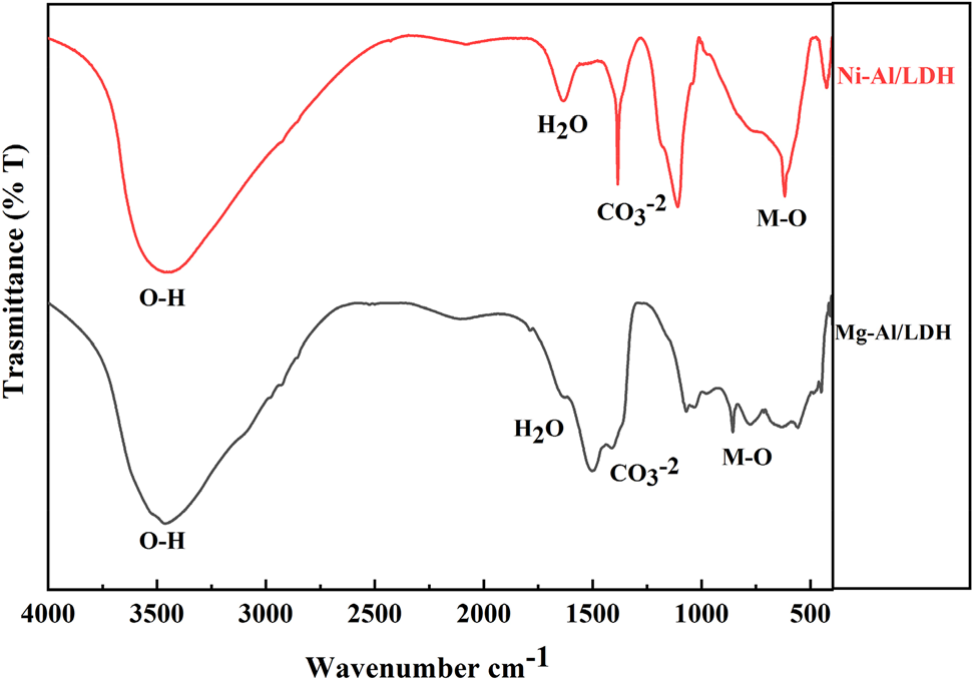
FT-IR spectra of Mg-Al/CO_3_ LDH (black line) and Ni-Al/CO_3_ LDH (red line) synthesised by co-precipitation method at PH 11.

The infrared absorption spectroscopy is another useful tool for the characterization of LDHs, involving the vibrations in the octahedral lattice, the hydroxyl groups and the interlayer anions. Fig. 3 presents the FT-IR spectrum of Mg-Al/CO_3_ LDH and Ni-Al/CO_3_ LDH both shows the absorption band around 3500 cm^−1^ can be assigned to the stretching vibration of the hydroxyl groups of LDH layers and interlayer water molecules. The bending mode of water molecules is responsible for the weak band at 1644 cm^−1^. The band with maximum peak at 1384 cm^−1^ belongs to stretching vibration of carbonate ions intercalated between the layers. Finally, the bands at 1100 cm^−1^, and 563 cm^−1^ can be attributed to M–O stretching modes and M–O–H bending vibrations.

**Fig. 4:**
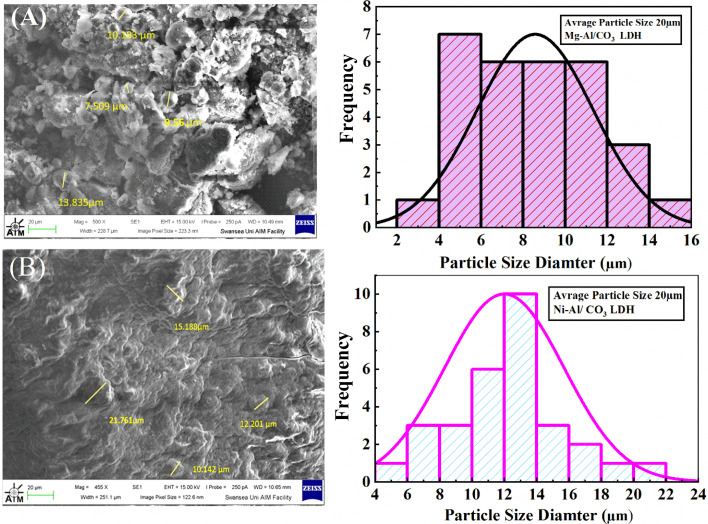
Scanning electron microscopy (SEM) images (A) of Mg-Al/CO_3_ LDH (B) Ni-Al/CO_3_ LDH synthesised by co-precipitation method at pH 11.

Fig. 4 presents the scanning electron microscopy (SEM) images (a) of Mg-Al/CO_3_ LDH (b) Ni-Al/CO_3_ LDH The SEM micrograph of the Mg-Al/CO_3_ layered double hydroxide (LDH) synthesized by co-precipitation reveals a well-developed plate-like morphology, characteristic of LDH structures. The layers appear as thin, aggregated nanosheets with relatively uniform size and a stacked or rosette-like arrangement. The approximate sizes of the particles fall in the 6–12 μm range. This morphology indicates effective crystal growth and layered assembly under controlled pH conditions, promoting the formation of stable, lamellar LDH structures.

In contrast, the SEM image of Ni-Al/CO_3_ LDH shows a more irregular and less defined platelet structure. The particles are smaller, with a more clustered and compact appearance. The approximate sizes of the particles fall in the 10–16 μm range. This suggests that the substitution of Mg^2+^ with Ni^2+^ influences the nucleation and growth behavior, possibly leading to a lower crystallinity or more disordered aggregation of the LDH nanosheets.^32^ The difference in ionic radius and coordination preference between Ni^2+^ and Mg^2+^ may account for the variation in morphology.

**Fig. 5:**
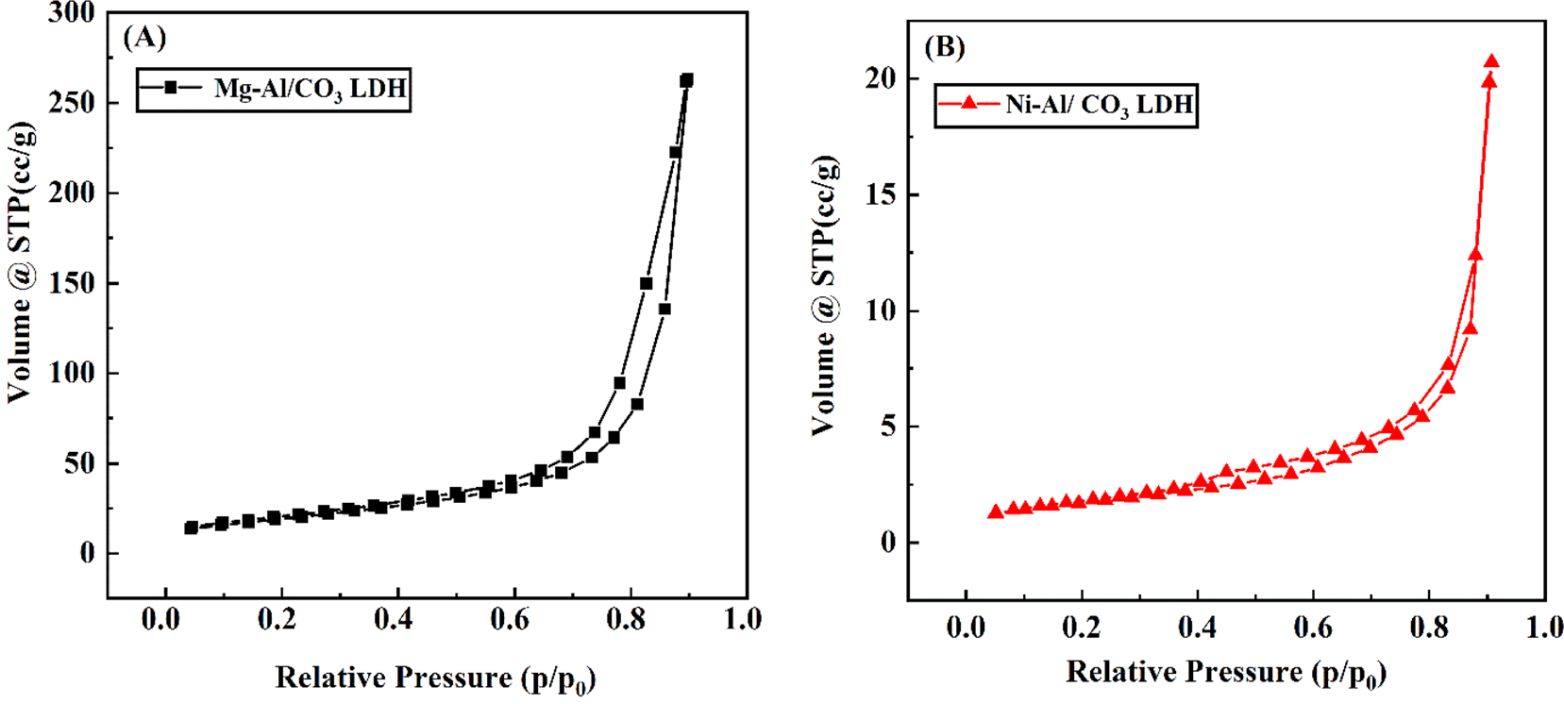
Nitrogen adsorption/desorption isotherm of: (A) of Mg-Al/CO_3_ LDH (B) Ni-Al/CO_3_ LDH.

The N_2_ adsorption–desorption curves over the samples are shown in Fig. 5. The curves follow from type III isotherm, indicating mesoporous materials. Table 1 shows the data of the N_2_ adsorption–desorption isotherm study was carried out using BET, BJH, and Langmuir techniques. According to BET analysis, the specific surface area of the Mg-Al/CO_3_ LDH and Ni-Al/CO_3_ LDH determined were 82.637 and 5.95 m^2^ g^−1^, respectively. In addition, the average pore size and pore volume of Mg-Al LDH were 17.6 nm and 0.435 cm^3^ g^−1^, whereas the average pore size and pore volume of Ni-Al LDH were determined 19.39 nm and 0.033 cm^3^ g^−1^.

**Table 1:** A brief overview of the N_2_ adsorption–desorption isotherm study was carried out using BET, BJH, and Langmuir techniques

The physisorption characteristic		Mg–Al	Ni–Al
BET summary	Specific surface area (m^2^ g^−1^)	82.637	5.950
BJH adsorption and desorption summary	Surface area (m^2^ g^−1^)	121.261	8.556
	Pore volume (cm^3^ g^−1^)	0.435	0.033
	Pore diameter (nm)	17.660	19.393
Langmuir summary	Surface area (m^2^ g^−1^)	472.205	125.854

### 3.2 Effect of contact time

**Fig. 6:**
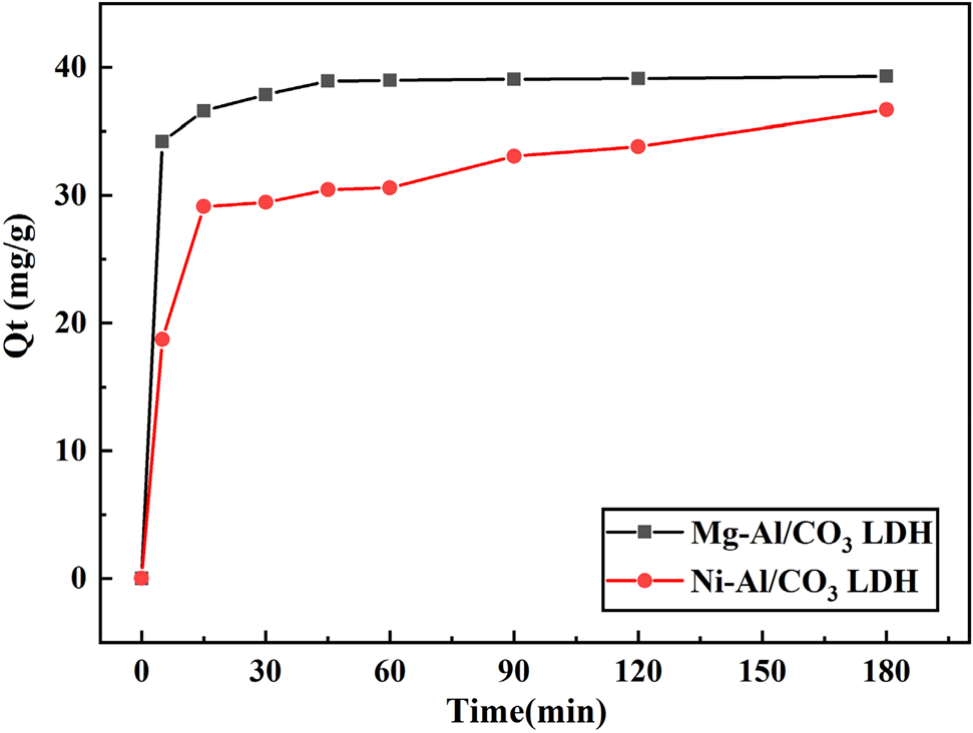
Plot between amount of adsorption 80 mg L^−1^ aniline blue dye on Mg-Al/CO_3_ LDH and Ni-Al/CO_3_ LDH with interval time.

The effect of contact time on the adsorption of aniline blue dye onto Mg-Al/CO_3_ LDH and Ni-Al/CO_3_ LDH in aqueous solution was investigated. As illustrated in Fig. 6, dye adsorption gradually increased with time and reached a maximum at 45 minutes, achieving a removal efficiency of approximately 85.00%. After this point, adsorption equilibrium was attained. Over time, dye molecules accumulate on the high-energy adsorption sites, eventually saturating them and restricting further diffusion into the adsorbent matrix.^33^ This saturation reduces the benefit of prolonged contact time, as the filled pores impede the adsorption of additional dye molecules.

### 3.3 Kinetic studies

The adsorption kinetics of aniline blue dye onto the surfaces of Mg-Al/CO_3_ LDH and Ni-Al/CO_3_ LDH were examined. In this study, two kinetic models—the Lagergren pseudo-first-order and pseudo-second-order models—were applied to analyze the adsorption behavior of aniline blue on the LDH adsorbents.^32^ The Lagergren model is among the most commonly employed equations for describing the adsorption rate of a solute from liquid solutions. The pseudo-first-order kinetic model is mathematically represented as follows:2
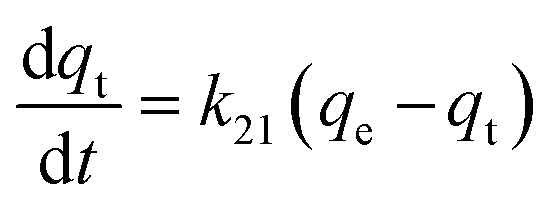

3 *q*_t_ = *q*_e_(1 − e^*kt*^)

**Fig. 7:**
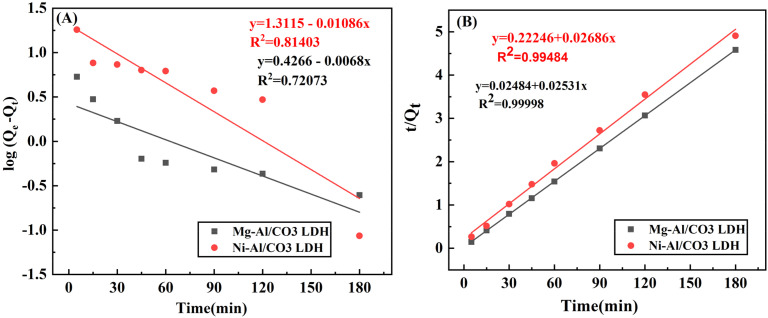
(A) Pseudo-first-order and (B) Pseudo-second-order models for the adsorption of aniline blue dye on the surfaces of Mg-Al/CO_3_ LDH and Ni-Al/CO_3_ LDH in pH 4 at 25 °C.

Or can be written as straight line equation4
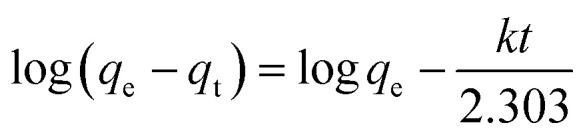
where *q*_t_ is the amount of ion adsorbed in mg g^−1^ at time (*t*), *q*_e_ is the maximum adsorption capacity, and *k* is the first-order rate constant (min^−1^). Fig. 7A, shows the plot of log(*q*_e_ − *q*_t_) *versus* time (*t*) gives a slope that is equal to −*k*_1_/2.303 and an intercept that is equal to determine the adsorption capacity and adsorption rate constants for aniline blue dye on LDHs adsorbent.

The second-order kinetics of adsorption proposed by Ho.^33^5
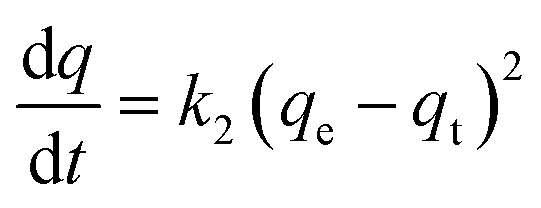
6
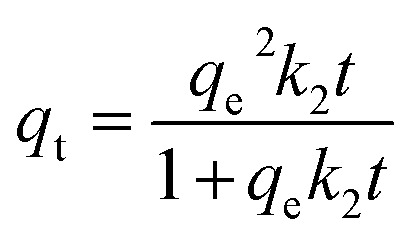
7
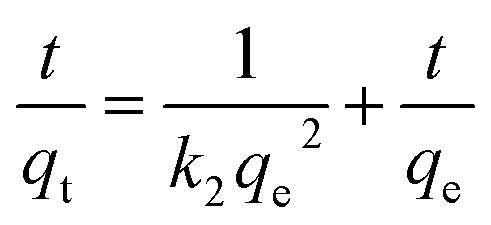


Fig. 7B, shows the plot of 
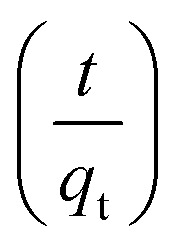
*versus* time (*t*) gives a slope that is equal to 
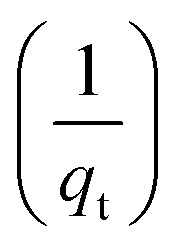
 and an intercept that is equal 
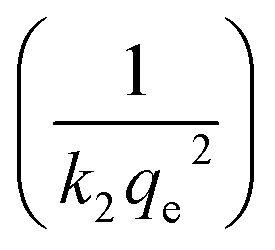
 to determine the adsorption capacity and adsorption rate constants for aniline blue dye on LDHs adsorbent.

The linear rate relationships and corresponding parameters for the kinetic models are presented in Table 2. Rate constants were determined from the slopes and equations of the trend lines associated with each model. The kinetic analysis demonstrated that both the pseudo-first-order (Fig. 7A) and pseudo-second-order (Fig. 7B) models effectively represented the adsorption behavior, as evidenced by high correlation coefficient (*R*^2^) values. However, the pseudo-second-order model showed a better fit, with *R*^2^ values of 0.99998 for aniline blue adsorption onto Mg-Al/CO_3_ LDH and 0.99484 for adsorption onto Ni-Al/CO_3_ LDH. Additionally, the close agreement between experimental and theoretical equilibrium adsorption capacities (*q*_e_) confirmed the model's high accuracy and reliability in describing aniline blue adsorption on both LDH types.^34^ Based on this model, the equilibrium adsorption capacities were determined to be 39.5 mg g^−1^ for Mg-Al/CO_3_ LDH and 37.23 mg g^−1^ for Ni-Al/CO_3_ LDH. The corresponding rate constants were 0.0258 g mg^−1^ min^−1^ and 0.0032 g mg^−1^ min^−1^, respectively, for an initial aniline blue concentration of 80 mg L^−1^.

**Table 2:** Kinetic parameters for the adsorption of 80 mg L^−1^ aniline blue dye on the surfaces of Mg-Al/CO_3_ LDH and Ni-Al/CO_3_ LDH

Adsorbent	Pseudo first order				Pseudo second order		
	*q*_max_ (mg g^−1^)	*K*_1_ (min^−1^)	*q*_e_ (mg g^−1^)	*R* ^2^	*K*_2_ g mg^−1^ min^−1^	*q*_e_ (mg g^−1^)	*R* ^2^
Mg-Al/CO_3_ LDH	39.3	0.0156	2.670	0.72073	0.0258	39.5	0.99998
Ni-Al/CO_3_ LDH	36.69	0.0250	20.48	0.81403	0.0032	37.23	0.99484

### 3.4 Adsorption isotherms

The experimental data have been analyzed by the linear forms of the Freundlich and Langmuir model isotherms represented by the following equations.

Freundlich isotherm;9
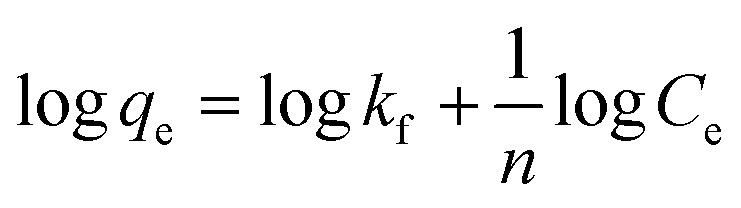
where, *q*_e_ amount of adsorbed at equilibrium mg g^−1^, *C*_e_ concentration at equilibrium mg L^−1^, *K*_f_ Freundlich constant refers to the adsorption capacity, *n* Freundlich constant related to the intensity of adsorption. The plot log *q*_e_ against log *C*_e_ gives a slope equal to (1/*n*), with intercept equal to log *k*_f_ and Langmuir isotherm is equal to:10
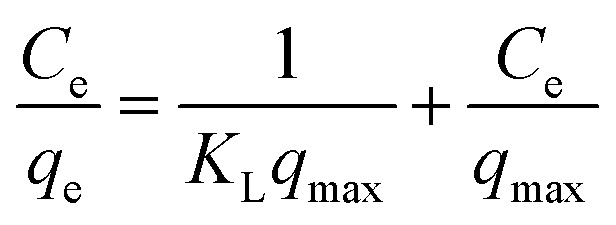
where, *q*_e_ amount of adsorption at equilibrium mg g^−1^, *C*_e_ the concentration of adsorbed at equilibrium mg L^−1^*K*_L_ = Langmuir constant related to adsorption capacity mg g^−1^, *q*_max_ Langmuir constant related to intensity of adsorption *L* mg^−1^. The plot (*C*_e_/*q*_e_) against *C*_e_ it gives a straight line producing a slope equal to (1/*q*_max_) with intercept equal to (1/*K*_L_*q*_max_).

**Fig. 8:**
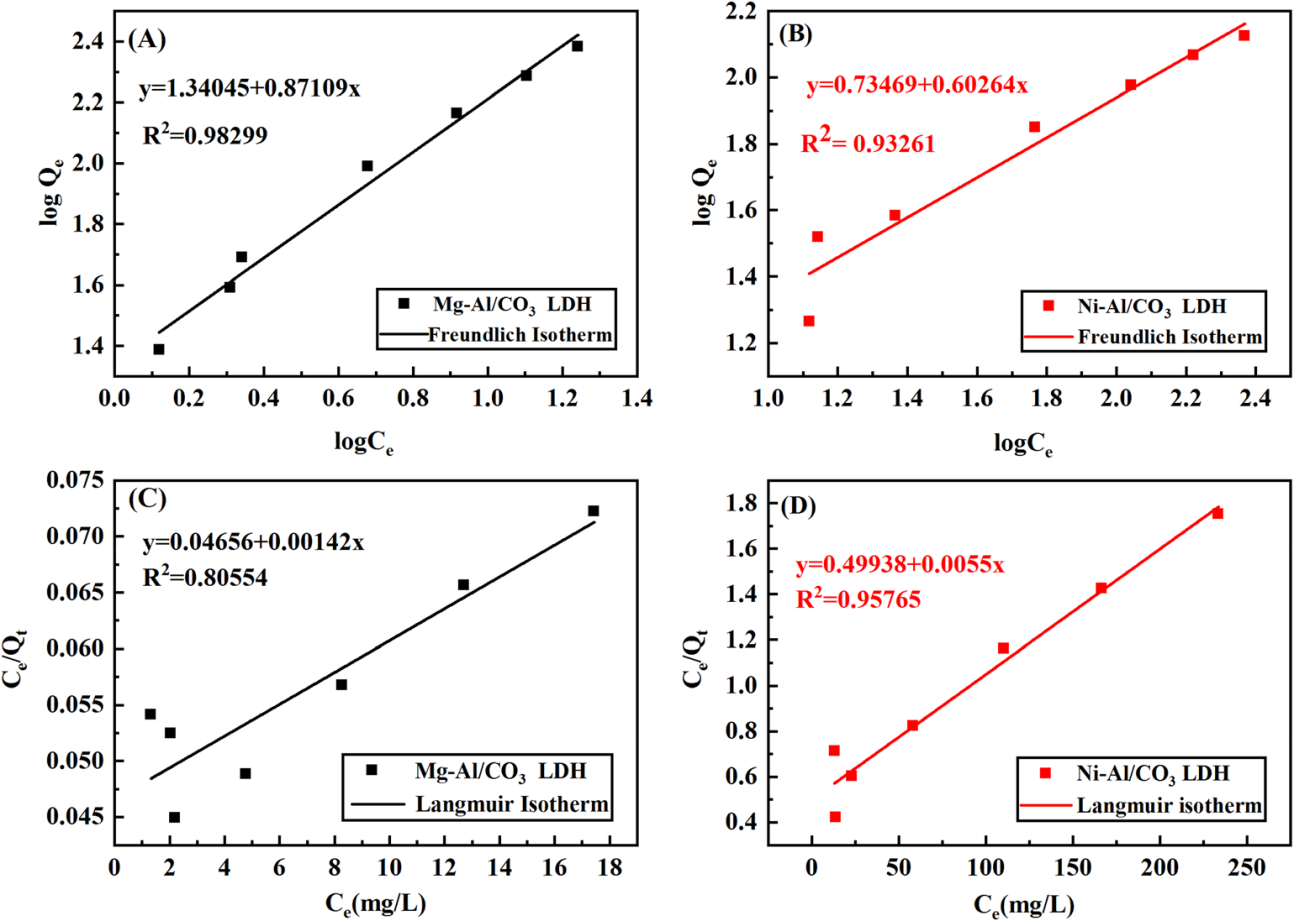
Adsorption isotherms plot for the adsorption of aniline blue dye (A) Freundlich's adsorption isotherm onto Mg-Al/CO_3_ LDH (B) Freundlich's adsorption isotherm onto Ni-Al/CO_3_ LDH (C) Langmuir's adsorption isotherm onto Mg-Al/CO_3_ LDH and (D) Langmuir's adsorption isotherm onto Ni-Al/CO_3_ LDH.

Adsorption isotherms are essential tools for understanding the nature of adsorption processes, providing insights into the affinity between adsorbate and adsorbent, the mode of adsorption (whether it occurs in a monolayer or multilayer), and the overall adsorption capacity. Fig. 8 shows the adsorption isotherms for aniline blue on Mg-Al/CO_3_ LDH and Ni-Al/CO_3_ LDH. The findings reveal that the quantity of dye adsorbed rises swiftly with increasing dye concentration before leveling off at a plateau, indicating saturation of the available adsorption sites and suggesting monolayer adsorption behavior. To interpret the equilibrium adsorption data, two widely adopted isotherm models Langmuir and Freundlich isotherms were applied. The Langmuir model assumes adsorption on a homogenous surface with identical energy across all active sites.^35^Table 3 summarizes the parameters derived from both models. For Mg-Al/CO_3_ LDH, the Freundlich model yielded a higher maximum adsorption capacity and a stronger correlation coefficient (*R*^2^ = 0.982) than the Langmuir model (*R*^2^ = 0.805), indicating that multilayer, physical adsorption dominated due to dye aggregation on the adsorbent surface. In contrast, for Ni-Al/CO_3_ LDH, the Langmuir model provided the best fit, with a higher correlation coefficient (*R*^2^ = 0.957) compared to the Freundlich model (*R*^2^ = 0.932), suggesting monolayer adsorption on a uniform surface with energetically equivalent sites.

**Table 3:** Langmuir and Freundlich isotherms adsorption of aniline blue dye on the surfaces of Mg-Al/CO_3_ LDH and Ni-Al/CO_3_ LDH in pH4

Adsorbents	Langmuir isotherm			Freundlich isotherm		
	*q*_max_ (mg g^−1^)	*K*_L_ (L mg^−1^)	*R* ^2^	*K*_f_ (1/g)	1/*n*	*R* ^2^
Mg-Al/CO_3_ LDH	704	0.0305	0.8055	21.9	0.871	0.9829
Ni-Al/CO_3_ LDH	181	0.011	0.9576	5.43	0.602	0.9326

### 3.5 Effect of pH

**Fig. 9:**
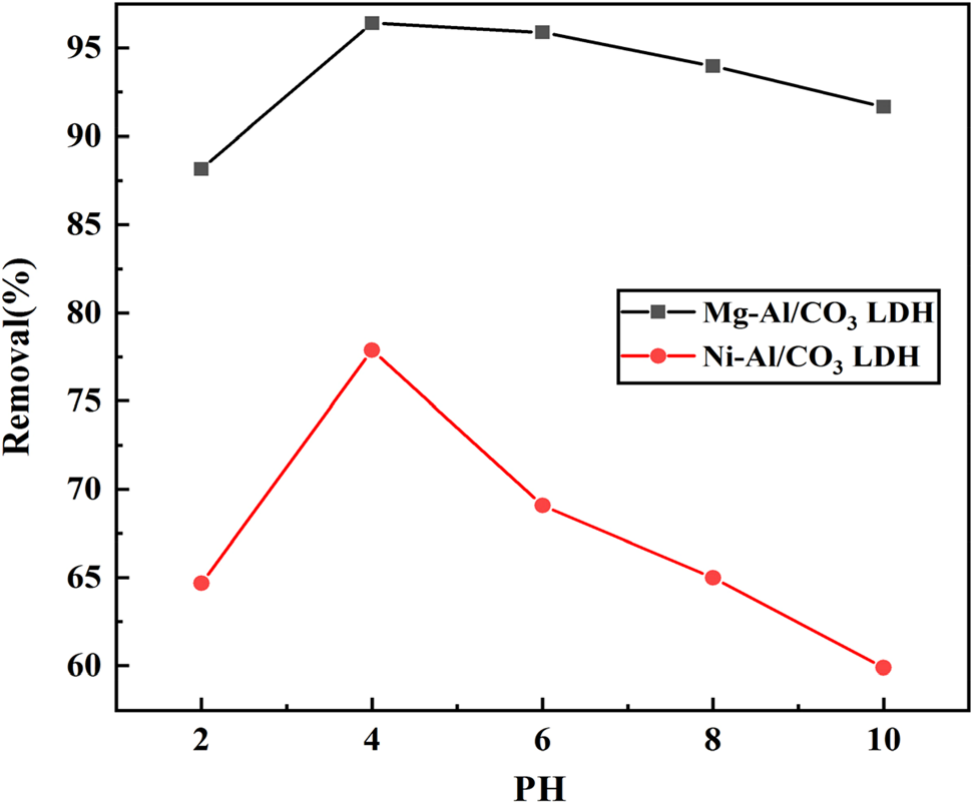
Effect of pH on the adsorption of adsorption 80 mg L^−1^ aniline blue dye on Mg-Al/CO_3_ LDH and Ni-Al/CO_3_ LDH at 25 °C.

The pH value of the dye solution can affect both the surface charge of the adsorbent and the ionization of the dye molecules, which significantly impacts their interaction. In this study, the influence of initial pH on adsorption was examined at 25 °C using a dye concentration of 80 mg g^−1^, within a pH range of 2 to 10, as illustrated in Fig. 9. It was found that the adsorption efficiency of the cationic dye aniline blue gradually decreased as the pH increased, with the lowest efficiency observed at pH 10. The highest adsorption performance occurred at pH 4, likely due to the increased H^+^ ion concentration under acidic conditions, which resulted in a more positively charged adsorbent surface. This positive surface charge enhanced electrostatic attraction between the adsorbent and aniline blue, leading to greater dye uptake. The strong interaction is attributed to the electrostatic attraction between the cationic dye and the negatively charged Mg-Al/CO_3_ LDH and Ni-Al/CO_3_ LDH materials.

### 3.6 Adsorption thermodynamics

**Fig. 10:**
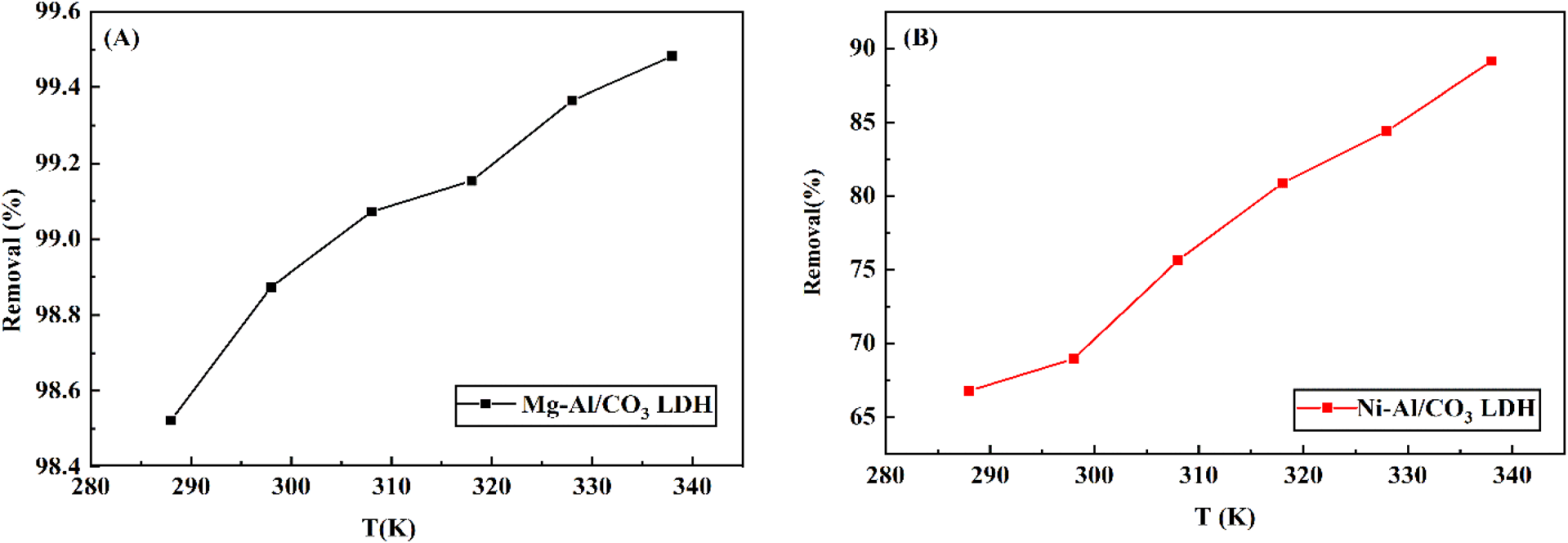
Effect of temperature on the aniline blue uptake (initial concentration = 80 mg L^−1^, pH = 4) on the surface of (A) Mg-Al/CO_3_ LDH (B) Ni-Al/CO_3_ LDH.

The thermodynamic characteristics of adsorption are commonly represented through adsorption isotherms, including models like Langmuir and Freundlich, which express the relationship between the quantity adsorbed and either pressure or concentration at a fixed temperature. Since adsorption is influenced by temperature, it allows for straightforward evaluation of thermodynamic parameters.^36^ Considering thermodynamics is essential in adsorption studies to determine whether the process is spontaneous and viable. Adsorption tests were conducted across a range of temperatures (288, 298, 308, 318, 328, and 338 K) to evaluate the impact of thermal variation on aniline blue removal efficiency, as presented in Fig. 10. The graph reveals increasing in dye adsorption with rising temperature. Specifically, the removal efficiency of aniline blue on Mg-Al/CO_3_ LDH increased from 94.5% to 99.5% as the temperature increased from 15 °C to 65 °C. Similarly, for Ni-Al/CO_3_ LDH, the efficiency increased from 66.4% to 87.5% over the same temperature range.

Consequently, experimental adsorption data are utilized to calculate key thermodynamic parameters, namely the standard Gibbs free energy change (Δ*G*° in KJ mol^−1^), standard entropy change (Δ*S*° in J K^−1^ mol^−1^), and standard enthalpy change (Δ*H*° in KJ mol^−1^), using the following equations:11
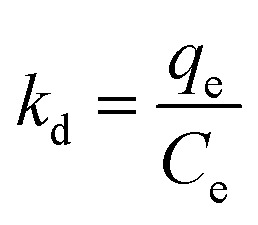
12
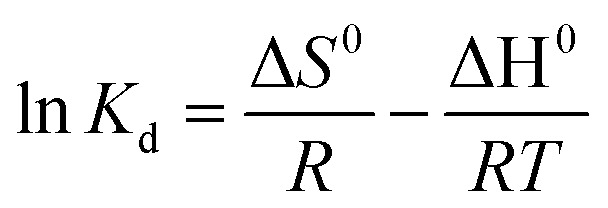

13 Δ*G*° = Δ*H*° − *T*Δ*S*

where *R* is the universal gas constant, *K*_d_ is a thermodynamic equilibrium constant, and *T* (K) is the absolute solution temperature. The distribution coefficient (*K*_d_) were calculated at different temperatures using eqn (11) and data are listed in Table 4. While the standard free energy (Δ*G*°) evaluated using eqn (12).

**Table 4:** The data of *K*_d_ value at different temperatures of aniline blue adsorption on Mg-Al/CO_3_ LDH and Ni-Al/CO_3_ LDH

Adsorbent	*K*_d_				
	*T* = 288 K	*T* = 298 K	*T* = 308 K	*T* = 318 K	*T* = 338 K
Mg-Al/CO_3_ LDH	33.33	43.89	53.39	78.26	96.11
Ni-Al/CO_3_ LDH	1.01	1.11	1.5	2.71	4.11

**Fig. 11:**
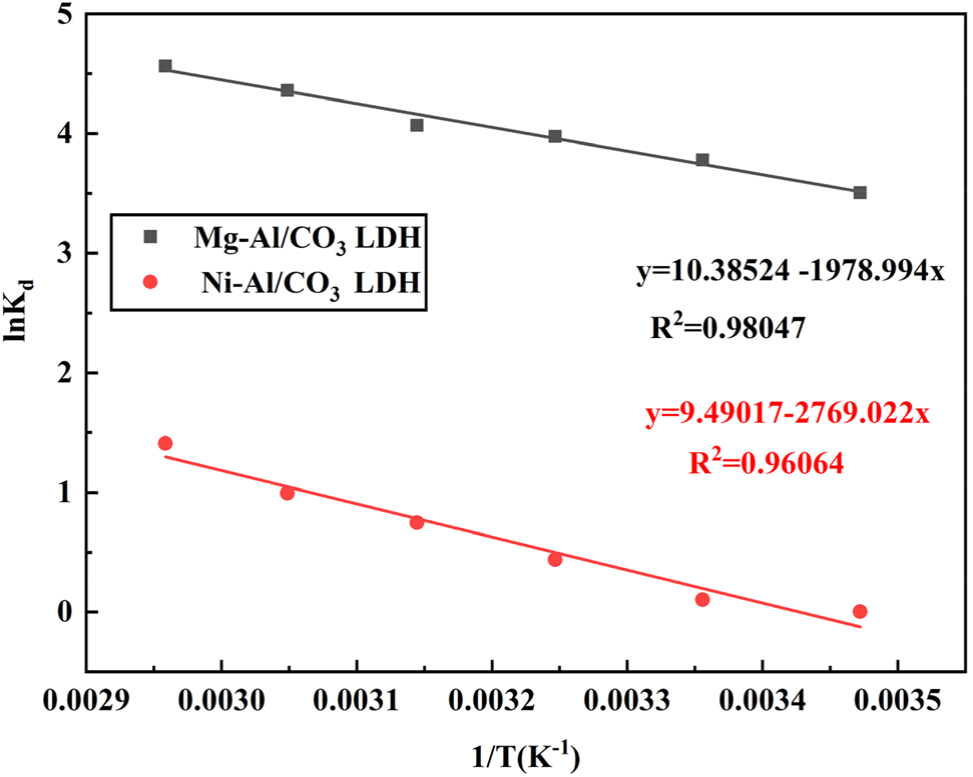
Graphical representation of ln *K*_d_ as a function of 1/*T* illustrating the temperature-dependent adsorption behavior of aniline blue dye on Mg-Al/CO_3_ and Ni-Al/CO_3_ layered double hydroxides.

The enthalpy (Δ*H*°) and entropy (Δ*S*°) of adsorption were calculated from the slope and intercept of the linear curve of ln *K*_d_*vs.* 1/*T*, respectively as shown Fig. 11.

The thermodynamic parameters including the standard Gibbs free energy change (Δ*G*°, kJ mol^−1^), standard entropy change (Δ*S*°, J K^−1^ mol^−1^), and standard enthalpy change (Δ*H*°, kJ mol^−1^) were computed and are summarized in Table 5. As observed, the negative values of Δ*G*° across all studied temperatures Δ*G*° is negative indicating that the adsorption of aniline blue on Mg-Al/CO_3_ LDH is spontaneous across the entire temperature range. The increasing negativity of Δ*G*° with temperature confirms the endothermic nature of the process. While for Ni-Al/CO_3_ LDH, at lower temperatures (*T* = 288 K), Δ*G*° is slightly positive, indicating non-spontaneous adsorption. As temperature increases, Δ*G*° becomes negative, showing the process becomes spontaneous only at higher temperatures. This confirms that adsorption on Ni-Al/CO_3_ LDH is more strongly temperature-dependent.

**Table 5:** Thermodynamic parameters data at different temperatures of aniline blue adsorption on Mg-Al/CO_3_ LDH and Ni-Al/CO_3_ LDH

Adsorbent	Δ*S*° (J K^−1^ mol^−1^)	Δ*H*° (kJ mol^−1^)	Δ*G*° (kJ mol^−1^)					
			*T* = 288 K	*T* = 298 K	*T* = 308 K	*T* = 318 K	*T* = 328 K	*T* = 338 K
Mg-Al/CO_3_ LDH	86	16.45	−8.318	−9.178	−10.038	−10.898	−11.758	−12.618
Ni-Al/CO_3_ LDH	7.89	23.02	0.3068	−0.4922	−1.2812	−2.0702	−2.564	−3.6482

Moreover, the positive Δ*H*° values indicate that the adsorption process is endothermic, implying that higher temperatures enhance the dye uptake. The positive Δ*S*° values further imply increased disorder at the solid–liquid interface, reflecting an improvement in the adsorption mechanism's randomness during the interaction between aniline blue and the LDH materials.^37^

### 3.7 Comparison between Mg-Al/CO_3_ LDH and Ni-Al/CO_3_ LDH in the adsorption of aniline blue dye

The differences in sorption capacities between Mg-Al/CO_3_ LDH and Ni-Al/CO_3_ LDH, as demonstrated by the data in Tables 1, 2, 3, and 5, can be attributed to various structural and physicochemical factors. Mg-Al/CO_3_ LDH exhibits markedly superior adsorption performance, which is primarily due to its significantly greater surface area and pore volume. These attributes enhance the accessibility and interaction of dye molecules with the adsorbent surface, thereby promoting more efficient physisorption compared to Ni-Al/CO_3_ LDH, which possesses much lower values in these critical structural parameters. Moreover, the distinct electronic configurations and bonding behaviors of Mg^2+^ and Ni^2+^ ions may affect their interactions with aniline blue molecules. Additional differences in crystallinity, pore size distribution, and layer basicity further contribute to the observed variations in adsorption efficiency.

Kinetic analysis indicates that the pseudo-second-order model provides a better fit for the adsorption behavior of both LDHs, suggesting that chemisorption is the rate-limiting mechanism. Notably, Mg-Al/CO_3_ LDH has a higher rate constant (*K*_2_ = 0.0258 g mg^−1^ min^−1^) than Ni-Al/CO_3_ LDH (*K*_2_ = 0.0032 g mg^−1^ min^−1^), indicating faster and more efficient dye uptake.

The adsorption data modeled by both Langmuir and Freundlich isotherms also highlight substantial differences. The Langmuir model, which assumes monolayer adsorption on a uniform surface, reveals that Mg-Al/CO_3_ LDH has a much higher maximum adsorption capacity (*q*_max_ = 704 mg g^−1^) compared to Ni-Al/CO_3_ LDH (*q*_max_ = 181 mg g^−1^). Furthermore, the Langmuir constant (K^L^) is higher for Mg-Al (0.0305 L mg^−1^) than for Ni-Al (0.011 L mg^−1^), indicating stronger binding affinity toward aniline blue.

Thermodynamic parameters (Δ*G*°, Δ*H*°, and Δ*S*°) provide further insight into the sorption mechanisms. Mg-Al/CO_3_ LDH shows greater thermodynamic favorability, as evidenced by more negative Gibbs free energy values (Δ*G*°), a moderately endothermic enthalpy change (Δ*H*°), and notably higher entropy (Δ*S*°). These results confirm the more spontaneous and efficient adsorption process in Mg-Al/CO_3_ LDH compared to Ni-Al/CO_3_ LDH.

### 3.8 Mechanism of adsorption of aniline blue on Mg-Al/CO_3_/LDH and Ni-Al/CO_3_ LDH

**Fig. 12:**
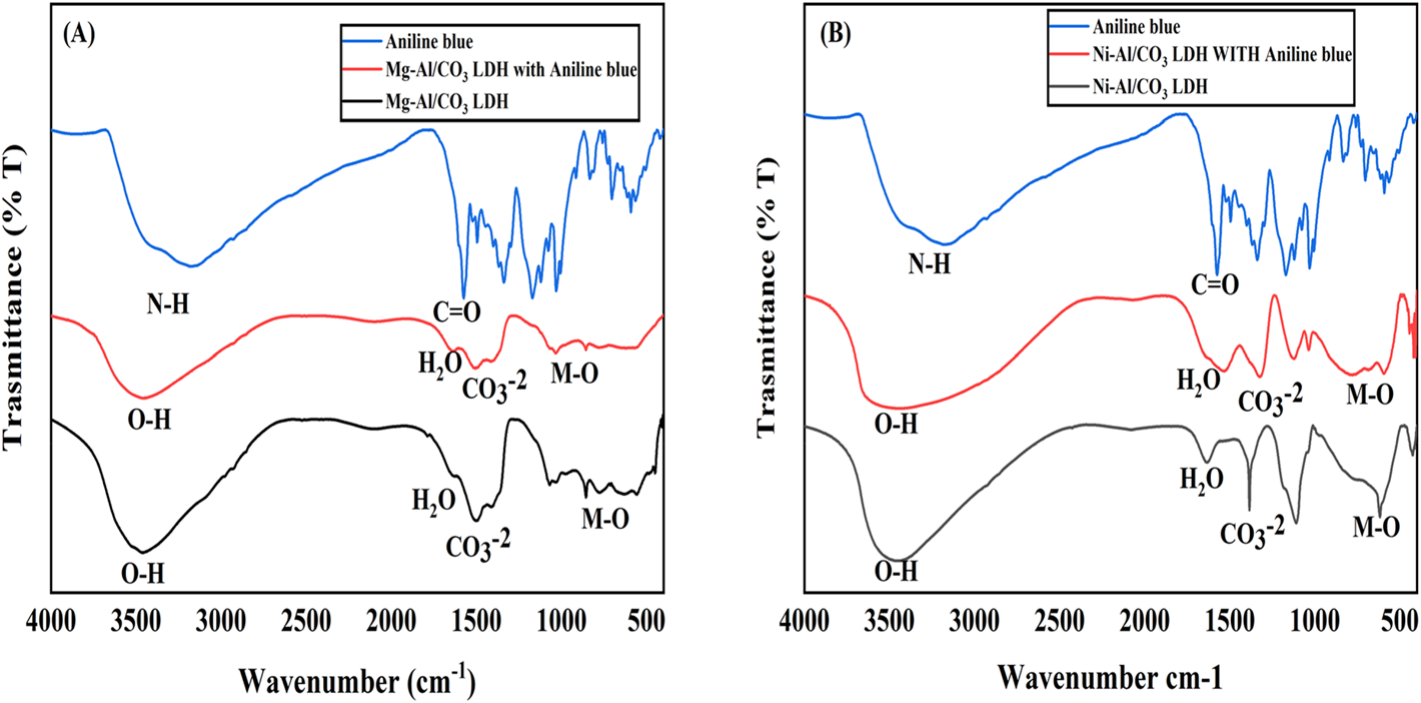
FT-IR spectrum of aniline blue dye before adsorption (blue line), LDH after adsorption (red line) and LDH before adsorption (black line) (A) Mg-Al/CO_3_/LDH and (B) Ni-Al/CO_3_ LDH.

Based on the FTIR spectra in the provided Fig. 12, the adsorption mechanism of aniline blue dye onto Mg-Al/CO_3_ LDH and Ni-Al/CO_3_ LDH can be inferred by analyzing the shifts, appearance, or disappearance of characteristic peaks after adsorption. The FT-IR of LDHs before adsorption shows broad O–H stretching around 3400 cm^−1^ due to interlayer water and hydroxyl groups.^38^ The peak H_2_O bending appear at 1640 cm^−1^ and CO_3_^−2^ stretching vibrations at 1350–1400 cm^−1^. While the peak of M–O and M–OH vibrations appear below 800 cm^−1^. After adsorption aniline blue on LDHs a new or shifted bands corresponding to AB functional groups appear or overlap in the LDH spectrum, confirming adsorption. A broadening of O–H band and slight shift indicates H-bonding between AB and surface (OH) groups. The appearance of C=O and N–H peaks from aniline blue suggests its incorporation.^39^ The intensity of CO_3_^−2^ may reduce or shift, indicating partial exchange with dye anions or surface interaction. In addition, the peak of M–O region (500–800 cm^−1^) shows minor changes, indicating electrostatic interaction or complexation.^40^

The results indicate successful adsorption of aniline blue onto both Mg-Al/CO_3_ and Ni-Al/CO_3_ LDHs through a combination of electrostatic attraction, hydrogen bonding, and surface interaction. The Mg-Al/CO_3_ LDH shows more significant spectral changes, supporting its higher adsorption capacity and stronger interaction with the dye compared to Ni-Al/CO_3_ LDH.

## 4 Conclusion

The study demonstrates that Mg-Al/CO_3_ and Ni-Al/CO_3_ LDHs are promising, low-cost materials for the effective adsorption of aniline blue dye from aqueous solutions. Their adsorption behavior is best described by the Freundlich isotherm and pseudo-second-order kinetics, indicating multilayer physical adsorption and chemisorption dynamics. Thermodynamic analyses confirm the process is spontaneous and endothermic, with increased entropy at the solid–liquid interface. These characteristics highlight the suitability of these LDHs for application in wastewater treatment technology.

## Author contributions

Material preparation, data collection and analysis and writing were performed by [kareem and Kareem Al-Salihi, Arman Ameen kaka Mhamad, Rebaz Fayaq HamaRawf]. The authors read and approved the final manuscript.

## Conflicts of interest

The authors have no relevant financial or non-financial interests to disclose.

## Data Availability

The author confirms that the data supporting the outcomes of this research are accessible within the article.
